# The Association between Serum LDL Cholesterol and Genetic Variation in Chromosomal Locus 1p13.3 among Coronary Artery Disease Patients

**DOI:** 10.1155/2015/678924

**Published:** 2015-03-08

**Authors:** Nasser M. Rizk, Ayman El-Menyar, Huda Egue, Idil Souleman Wais, Hissa Mohamed Baluli, Khalid Alali, Fathi Farag, Noura Younes, Jassim Al Suwaidi

**Affiliations:** ^1^Health Sciences Department, CAS, Qatar University, P.O. Box 2713, Doha, Qatar; ^2^Cardiology Unit, Ahmed Maher Teaching Hospital, Cairo, Egypt; ^3^Clinical Medicine, Weill Cornell Medical School, P.O. Box 24144, Doha, Qatar; ^4^Cardiology Unit, Al-Emadi Hospital, P.O. Box 5804, Doha, Qatar; ^5^Clinical Chemistry Department, Hamad Medical Corporation (HMC), P.O. Box 3050, Doha, Qatar; ^6^Cardiology Department, Heart Hospital, Hamad Medical Corporation (HMC), P.O. Box 3050, Doha, Qatar

## Abstract

*Background*. Several polymorphisms of a locus on chromosome 1p13.3 have a significant effect on low-density lipoprotein cholesterol (LDL-C), atherosclerosis, and acute coronary syndrome (ACS).* Methods*. We aimed to investigate the association between rs599839, rs646776, and rs4970834 of locus 1p13.3 and serum LDL-C and severity of coronary artery stenosis in ACS patients. Genotyping of the rs599839, rs646776, and rs4970834 polymorphisms was performed on Arab patients undergoing coronary angiography for ACS. Patients were divided into group A (ACS with insignificant stenosis (<50%)) and group B (with significant stenosis (≥50%)).* Results*. Patients carrying the minor G allele in rs599839 had significantly lower mean of LDL-C (2.58 versus 3.44 mM, *P* = 0.026) than homozygous A allele carriers (GG versus AA). Carriers of minor C allele in rs64776 had significantly higher mean of HDL-C (2.16 versus 1.36 mM, *P* = 0.004) than carriers of the T alleles (AA versus GG). The odd ratio and 95% confidence interval for dominant model for G allele carriers of rs599839 were 0.51 (0.30–0.92), *P* = 0.038, among patients with significant stenosis.* Conclusions*. Polymorphisms rs646776 and rs599839 of locus 1p13.3 were significantly associated with LDL-C and other lipid parameters. In addition, the G-allele carriers of variant rs599839 had a significant protective effect against the atherosclerosis.

## 1. Introduction

Cardiovascular disease (CVD) is a leading cause of morbidity and mortality worldwide [1]. Serum LDL cholesterol has a contributing role in the pathogenesis of cardiovascular diseases [2]. Clinical and epidemiological studies showed that increased levels of low-density lipoprotein cholesterol (LDL-C) promote premature atherosclerosis [3, 4]. Low-density lipoprotein cholesterol, the major carrier of blood cholesterol, has been implicated in the buildup of cholesterol in atherosclerotic plaques. Several mechanisms were postulated to explain the role of LDL in the pathogenesis of atherosclerosis. Oxidation of LDL-C has been shown to result in numerous changes in its biologic properties that could have pathogenetic importance, including accelerated uptake in macrophages, cytotoxicity, and chemotactic activity for monocytes in arterial walls [5, 6]. It has also been shown that subjects with small dense LDL-C particles show an impaired response to the endothelium-dependent vasodilator acetylcholine [7]. Experimental studies have demonstrated that decreasing serum LDL cholesterol concentrations have a clinical effect on the CVD morbidity and mortality [8]. Thus, regulation of LDL cholesterol represents an important target for developing additional interventional approaches to decrease the hazards of CVD.

Coronary artery disease (CAD) as a consequence of the atherosclerotic changes in coronary arteries may also result from the action of multiple genetic and environmental factors [9]. Genetic susceptibility to ischemic heart diseases (IHD) may be determined by specific polymorphic variants of genes encoding isoform involved in processes that are important in the pathogenesis of atherosclerosis.

Recently, genome-wide association studies (GWAS) reported a number of candidate loci conferring risk or protection from CAD and myocardial infarction (MI) [10, 11].

Recent genome-wide association studies (GWAS) have discovered a significant association of hypercholesterolemia and myocardial infarction with polymorphisms on human chromosome 1p13.3. There are four genes in this region proline/serine-rich coiled-coil protein 1 (PSRC1), cadherin, EGF LAG seven-pass G-type receptor 2 (CELSR2), myosin binding protein H-like (MYBPHL), and sortilin 1 (SORT1) [12].

An intracellular receptor for apolipoprotein (apo) B100 known as sortilin is encoded by SORT1. It interacts with apoB100 in the Golgi apparatus and accelerates the hepatic synthesis and export of apoB100-containing lipoproteins, and so it regulates the circulating level of LDL-C. It has been shown that knockout mouse of sortilin receptor reduces secretion of lipoproteins from the liver and reduce the hypercholesterolemia and atherosclerotic plaques development in LDL receptor-deficient animals. In contrast, overexpression of sortilin accelerates hepatic release of lipoproteins and increases the circulating LDL levels [13].

The promising candidate loci on chromosomal location 1p13.3 have been linked consistently to LDL-C in all genetic association studies that examined this parameter [11, 13–15], which is a well-established risk factor for CAD [12].

GWAS showed the following variants rs599839, rs646776, and rs4970834 on chromosomal location 1p13.3 as genetic determinants of LDL metabolic parameters [14–16]. It has been successfully replicated in several studies [15–17]; however, one study has shown no association with CAD [18]. These polymorphisms are located on 1p13.3 in an approximately 97 KB large block of linkage disequilibrium. The variant rs599839 located in the 3- untranslated region of the PSRC1 gene shows strong linkage disequilibrium with other SNPs in this LD block [12]. In particular, rs599839 is the SNP that has the highest correlation with CAD and LDL-C in most studies [14, 15, 19, 20]. The minor allele in European populations (G, MAF0.33 in HapMap CEU; http://www.ncbi.nlm.nih.gov/projects/SNP/) was found to be associated with a lower risk of CAD and lower levels of LDL-C.

There are currently no equivalent data of such relationship among Arab population that evaluated the polymorphisms of genes modulating LDL-C level and its impact on CAD. The aim of this study is to investigate the associations of the SNPs: rs599839, rs646776, and rs4970834 of 1p13.3 locus with (1) serum LDL cholesterol, (2) other lipid profile, and (3) its value as risk factors of CAD (with and without LDL) in a cohort of well-characterized consecutive patients presented with acute coronary artery syndrome (ACS) that underwent coronary angiography.

## 2. Methods

### 2.1. Subjects

A case-control association study was performed on 227 consecutive Arab patients, who had been admitted with ACS to coronary care unit at Hamad Medical Corporation (HMC). Cardiovascular risk factors such as hypertension, obesity, diabetes, and smoking were obtained. Blood samples were collected after 12 hours overnight fasting. The diagnosis of ACS was made by the attending physicians according to American College of Cardiology, American Heart Association (ACC/AHA) standards [21]. Type 2 diabetes was diagnosed according to the ADA criteria [22]. Dyslipidemia was identified based on the standards of the National Cholesterol Education Program Adult Treatment Panel III [23]. The cut-off points for body mass index (BMI) were used to define obesity according to world health organization “WHO” criteria [24].

All patients underwent coronary angiograms on the index admission. Two physicians assessed the degree of atherosclerotic lesions, and the number of coronary vessels affected. Males and females patients aged between 30 and 70 years were divided into two groups based on the extent of the atherosclerotic lesions, which were assessed by the angiographic data. The degree of atherosclerotic lesions and number of coronary vessels affected were assessed. Patients were categorized according to their coronary angiographic findings, that is, group A: with nonsignificant atherosclerotic lesion (<50% stenosis, insignificant lesion used as a control group, *n* = 91) and group B: atherosclerotic lesion causing luminal stenosis ≥50% (significant stenosis and used as a case group, *n* = 136) [25]. Individuals with diseases such as cancer, liver failure, renal failure, and autoimmune disorders were excluded. Human subjects data were extracted from patients recruited under protocols 12297 and 10043 that have been approved by the MRC, HMC.

## 3. Measurements

### 3.1. Clinical Measurements

Height, weight, waist circumference, and systolic and diastolic blood pressures were measured. Weight and height were measured in Kg and cm, respectively, down to one decimal point. Hypertension was a systolic and/or diastolic blood pressure ≥140 and/or ≥90 mmHg and/or a significant history of hypertension and/or under treatment of hypertension. The body mass index (BMI) was calculated by dividing the weight (Kg) over the square of height (m^2^).

### 3.2. Biochemical Assays

10 mL of peripheral blood was collected from each participating subject. 5 mL was used for DNA extraction, and the remaining 5 mL was used for determination of the biochemical parameters including plasma glucose, lipid profile, and cardiac markers including CK-MB and Troponin T. All biochemical assays were done at clinical chemistry labs at HMC-Qatar. Genetic analysis was performed in the Biomedical Research Labs of Qatar University.

### 3.3. Genotyping of the Polymorphisms of the Variants rs599839, rs646776, and rs4970834

The genotyping was performed as previously published [26]. In brief, DNA was extracted from whole blood samples of all study subjects, using the QiaAmp DNA blood Mini Kit Cat # 51360 (Qiagen, Germany) with RBC lysis solution Cat # 158904 (Qiagen, Germany). It was performed according to the manufacturer protocol. Measurements of DNA concentration and purity were done using the NanoDrop Spectrophotometer.

#### 3.3.1. Gene Amplification

**Figure 1:**
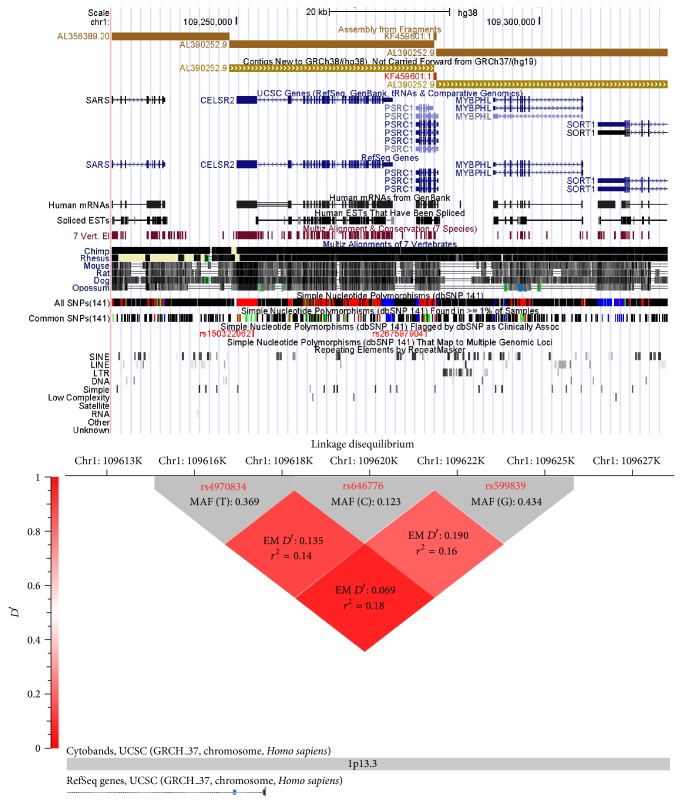
The genetic architecture of 1p13.3 and Linkage Disequilibrium (LD) of the rs599839, rs646776, and rs4970834 of on chromosomal location 1p13.3 in ACS patients. Red diamonds represent pairwise LD “EM* D*′” values between rs4970834 and rs646776 (upper left), rs646776 and rs599839 (upper right), and rs4970834, and rs599839 (lower), respectively.

rs599839, rs646776, and rs4970834 variants are located in chromosomal locus 1p13.3. The basis of the selection of theses SNPs was based on previous publications that indicated an association of these SNPs with the lipid profile and CVD [14, 17, 18], and it has an impact on the expression of* SORT1*, a nearby gene that mediates endocytosis and degradation of lipoprotein lipase [27].  Figure 1 shows the genetic architecture of 1p13.3 locus and the linkage disequilibrium (LD) between the 3 SNPs used in this study, with values representing *r* ^2^ values from hapview by golden helix software program. All SNPs were genotyped by allele-specific PCR, in which primers were designed to specifically amplify the reference allele. Polymorphisms of these SNPs were performed by the 5′ nuclease assay using TaqMan MGB probe by means of an ABI 7500 (Applied Biosystems, Foster City, CA). The primers and the probes of these SNPs polymorphisms were provided by the assay-on demand TM service by (Applied Biosystems). The 5′ nuclease assay was performed using 20 ng genomic DNA, 1x TaqMan Universal PCR Master Mix (Applied Biosystems), and 1x primer/probe mix using the proper conditions for amplifications by the manufacturer's instructions and negative controls were used.

### 3.4. Statistical Analysis

Data were explored for outliers, skewness, and normality and transformed when necessary if the normality assumption was violated. Continuous data are expressed as mean ± SD for normally distributed variables, median and interquartile range [25%–75%] for nondistributed continuous data, and number and percentage for categorical data. Two Student's* t*-tests and nonparametric Mann-Whitney evaluated the differences between continuous variables, and 2 independent samples* t*-tests were used accordingly for the analysis. Differences between categorical variables were evaluated by chi-square test. Genotype distributions and allele frequencies between the study groups were compared by constructing 2 × 2 contingency tables, *x* ^2^, and/or Fisher exact test corrected for Bonferroni adjustment for the number of SNPs. The Hardy-Weinberg equilibrium (HWE) was calculated using the chi-square test to determine genotype distribution in all study subjects. Odds ratios (ORs), 95% confidence intervals (CIs), and corresponding *P* values for the samples were analyzed by logistic regression analysis. ORs were computed using the minor homozygous alleles as the reference group unless otherwise stated. Pairwise linkage disequilibrium coefficients (*D*′) and haplotype frequency were estimated using the golden helix (SVS 8) software. Multiple testing corrections were applied to avoid false positive results such as with Bonferroni adjustments and false discovery rate using Golden Helix software. Estimated marginal means with 95% confidence interval of the lipid parameters in association with genotypes were evaluated utilizing general linear model adjusted for age, gender, BMI, type 2 diabetes, smoking, hypertension, and lipid-lowering therapy. Multiple step regression analysis was applied to assess the associations between the LDL (dependent variable) and the following independent variables: age, gender, BMI, diabetes, hypertension and dyslipidemia, and antilipidemic treatment and rs599839, rs646776, and rs4970834.

The biological interaction evaluates the alteration in the hazard associated with the contact to both factors: circulating LDL level and genotype. The biological interaction estimates the difference in the risk, which is expressed as OR (95% CI) when exposed to one factor alone or both factors together [28]. The interaction between the genotypes and the serum LDL-C was calculated using the biological approach [29]. The biological interaction estimates the difference in the risk associated with the exposure to both factors. The ratio between the risk observed in the presence of both factors and the risk observed in the reference group can be used to drive the synergy index (*S*) [30, 31].

All statistical analyses were performed using the SPSS program for Windows (version 21 statistical software, Texas Instruments, IL, USA), Graph Pad Prism (version 6, for Mac, Graph Pad Software, La Jolla California USA), and the Golden Helix SNP and Variation Suite (SVS 8, software; Bozeman, MT, USA) for genetic analysis. Two-tailed *P* value < 0.05 is statistically significant.

### 3.5. Power Calculations

We carried out a statistical power analysis using QUANTO software version 1.2 (http://hydra.usc.edu/gxe/_vti_bin/shtml.dll/request.htm) to ensure our sample size is sufficient to identify associations of our SNPs and their susceptibility with CAD/MI. Under the population parameter settings, prior data indicated that MAF of rs599839-G is 0.18 [32, 33] and setting of the odds-ratio of 0.55 and dominant model of inheritance [17], we need to study 226 subjects to be able to reject the null hypothesis that the rs599839-G rates for cases and controls are equal with probability (power) of 0.8. The type I error probability associated with this test of this null hypothesis is 0.05.

## 4. Results

### 4.1. Clinical, Biochemical, and Genetic Data of the Cardiac Patients

Tables 1(a) and 1(b) show the clinical and biochemical data collected from patients diagnosed with ACS based on the angiographic data of the coronary arteries. Among 227 subjects, 136 patients (60.0%) had significant stenosis (≥50%) and 91 patients (40.0%) had insignificant stenosis (<50.0%). Patients with significant stenosis (group B) had a mean age (SD) of 56.33 (10.59) years while patients with the insignificant stenosis (group A) had 56.36 (9.34) years, with *P* value = 0.701. Among patients with significant stenosis: 80.9% were males, 52.2% were obese, 61.7% were hypertensive, 62.5% were diabetic, 33.1% were dyslipidemic, and 46.3% were smokers. Among patients with insignificant stenosis: 74.7% were males, 49.5% were obese, 59.3% were hypertensive, 43.8% were diabetic, 45.2% were dyslipidemic, and 32.9% were smokers. Group (B) had a significantly higher frequency of diabetes and higher fasting blood glucose than group A (*P* = 0.008) and (*P* = 0.003), respectively. No significant difference was found between groups A and B for age; weight; BMI; gender; SBP; DBP; frequency of hypertension; dyslipidemia; smoking; lipid profile (TC, TG, HDL, and LDL); cardiac enzyme markers (troponin and CK-MB); and antidiabetic and statin treatment, as shown in Table 1.

In addition, overall the SNP genotypes and allelic frequencies were determined in all individuals. The call rate of all SNPs in this study was ≥90.0%, and genotype frequency did not deviate significantly from the Hardy-Weinberg Equilibrium (HWE) with *P* value >0.05.  Table 2 shows the genotype distribution of rs599839, rs646776, and rs4970834 polymorphisms in all subjects and according to the degree of the significant stenosis. There were a more frequent AA (48.4%) genotype for rs599839, TT (76.9%) genotype for rs646776, and CC (54.2%) genotype for rs4970834 among all study subjects.  Table 2 also presents the frequencies of minor alleles in each SNP among all study subjects: G (0.434) for rs599839, C (0.201) for rs646776, and T (0.369) for rs4970834. Utilizing the chi-square test with corrections for Bonferroni adjustments of SNPs numbers, only a significant association was observed between the minor allele (G) of rs599839 and the coronary artery stenosis *P* = 0.032, *χ* ^2^ = 3.68. The minor alleles of the other two SNPs rs646776 and rs4970834 were not significantly associated with a significant coronary artery stenosis among the study subjects as shown in Table 2.

#### 4.1.1. Linkage Disequilibrium (LD) of the Three SNPs (rs599839, rs646776, and rs4970834) in CAD Patients (Figure 1)

Pairwise LD between the 3 polymorphisms was evaluated using data from all subjects. Using the expectation-maximization (EM), the results of LD and the *r* ^2^ for all study subjects are presented in Figure 1. The results of the LD of the three pairs had low LD in the study subjects. Therefore, all SNPs rs599839, rs646776, and rs4970834 were included in the analysis (Figure 1).

### 4.2. Association with the Lipid Profile

#### 4.2.1. Associations of Genotypes with Lipid Parameters (Table 3)

[Table tab3]We assessed serum lipid concentrations (estimated marginal means with 95% CI) with respect to the genotypes of the variants rs599839, rs64776, and rs4970834 using different genetic models which were adjusted for age, gender, BMI, type 2 diabetes, smoking, hypertension, and antidiabetic and statin therapy. Patients carrying the G allele (GG versus AA) in rs599839 variant had significantly lower mean TC levels than A allele carriers (4.13 versus 5.29 mM, *P* = 0.041) and mean LDL-C (2.58 versus 3.44 mM, *P* = 0.026), respectively. Patients carriers of the C allele (CC versus TT) in rs64776 had significantly lower mean TG levels than T allele carriers (2.30 versus 6.69 mM, *P* = 0.018) and higher HDL-C compared to TT carriers (2.16 versus 1.15 mM, *P* = 0.004), respectively. The minor allele (T) of variant rs4970834 had no effect on any lipid parameter as shown in Table 3. The minor allele “G” of rs599839 was associated with a decreased LDL-C level of 0.2–0.3 mmol/L per allele, and with an increase by 0.08–0.18 mmol/L with HDL-C and with a decreased TC level of 0.23–0.98 mmol/L per allele. In addition, the minor allele C of rs646776 was also associated with an increase of HDL-level of 0.15–0.25 mmol/L per allele. The minor allele T of rs4970834 was not associated with a decreased LDL-C level of 0.33–0.58 mmol/L per allele and decreased 0.07–0.13 mmol/L with HDL-C.

#### 4.2.2. Associations between LDL-C, Genotype, and Cardiac Risk Factors Associated with the CAD of the Study Subjects (Table 4)

[Table tab4]Furthermore, we quantify the effect of the genotype and phenotypes associated with the risks of the CAD on the circulating LDL-C level. The stepwise regression analysis was performed to discriminate the significant effect of the following independent variables: age, gender, obesity, diabetic state, smoking, and statin treatment and rs599839, rs646776, and rs4970834 in their dominant mode on the dependent factor LDL-C among all CAD patients. The results show that the following independent variables have a statistically significant effect on LDL-C level, BMI (*P* = 0.028), diabetes (*P* = 0.005), and rs599839 (*P* = 0.014) as shown in Table 4 and contribute to 37.7% of LDL-C level among the study subjects. However, the regression analysis suffered from the problem of multicollinearity which is essentially a problem of correlated independent variables.

### 4.3. Association of rs599839, rs64776, and rs4970834 Genotypes with Angiographically Characterized CAD Patients

#### 4.3.1. Associations between SNPs Genotype and Degree of Coronary Stenosis (Table 5)

Table 5 presents the associations between the angiographic characters of coronary vessels stenosis with the variants rs599839, rs64776, and rs4970834 utilizing the logistic regression analysis. The stepwise regression models were used as follows: model 1 is unadjusted, model 2 is adjusted for age, gender, obesity, hypertension, diabetes, smoking, and family history of CAD, and model 3 for the cofounders adjusted in model 2 with LDL, HDL, and lipid-lowering treatment. Only the association between the G allele of rs599839 with CAD is significant even after adjustment for the cofounders counted in model 2 and model 3. The unadjusted odds ratio and 95% CI of the G allele of the variant rs599839 were 0.59 (0.34–0.97), *P* = 0.049 and after adjustment were 0.56 (0.32–0.94), *P* = 0.043 and 0.51 (0.30–0.92), *P* = 0.38 for models 2 and 3, respectively, indicating reduced risk for CAD stenosis.

**Figure 2:**
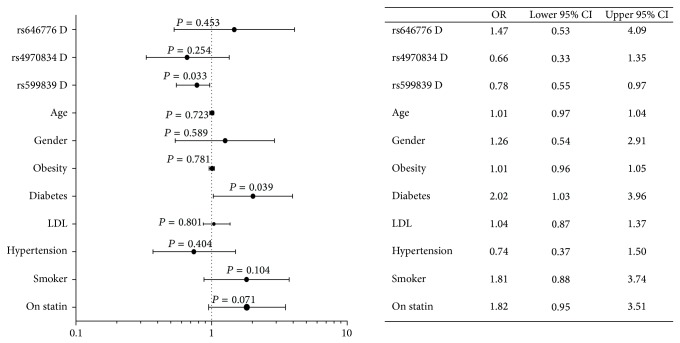
Logistic regression analysis of the factors involved as confounding factors on the response variable of the significant lesion (≥50%) versus nonsignificant stenosis (<50%) in all ACS patients. Data are presented as odds ratio and 95% CI. Data show the effects of CVD risk factors (genetic variants (in their dominant model)) in interaction with traditional CVD risks including the following variables: old age above 60, male gender, +obesity, +DM, +hypertension, +smoker, +hyper-LDL-C above desirable level, and statin treatment on severity of significant stenosis of the coronaries in the study subjects. Two-tailed* P* value is significant < 0.05.

We evaluated the possible assocaition of diabetes and statin with the variants rs599839, rs64776, and rs4970834 in ACS patients. We separated the ACS patients based on the presence and absence of diabetes and the use of statin or not. Using the same logistic regression models applied as above, the odds ratio were not significantly different between patients receiving statin and those not receiving statin therapy (data not shown). Next, we quantified the contribution of genetic SNPs (Dominant model; rare alleles carriers versus the common homozygous allele carriers) with the traditional CVD risks on the significant stenosis in ACS patients. These independent CVD factors included: old age above 60, male gender, obesity, DM, hypertension, smoking, serum LDL-C above the desirable level, and statin treatment. A logistic regression analysis was performed where these CVD factors were involved as independet factors to highlight its effect on the response variable (dependent) of the significant lesion (≥50%) versus the non-significant stenosis (<50%) in the ACS patients. The results show that diabetes increases the odds by 2.02 (1.03–3.96), *P* = 0.039 and carriers of the minor alleles of rs599839 decreases the risk with odds of 0.78 (0.55–0.97), *P* = 0.033 and this model may explain 17.8% of the model effect, as shown in Figure 2.

#### 4.3.2. Haplotype Frequency and Its Association with (Table 6)

[Table tab6]The alleles of the SNPs (rs599839, rs4970834, and rs646776) in order located in chromosomal locus 1p13.3 formed several frequent haplotypes with their associations, as shown in Table 6. Only the GTC haplotype formed by the rare alleles of the studied SNPs had a significant protective effect against the risk of the stenosis of coronary arteries in CAD with odds ratio 0.54 and 95% CI = 0.21–0.96. Its frequency in significant stenosis is nearly 1.8 times (29.5% versus 17.8%, *P* = 0.005) its frequency in nonsignificant stenosis, respectively. In addition, the other haplotypes formed had an insignificant effect on the risk of vessels stenosis, as shown in Table 6.

### 4.4. The Biological Interaction of High LDL-C and Genetic Variants at 1p13 Locos on Stenosis Severity of ACS Patients Expressed as Odds Ratio (OR) and 95% CI in ACS Patients (Table 7)

Given the causal association between high serum LDL and coronary stenosis in ACS, we tested the biological interaction between the genotypes and the serum level of LDL-C as an important player in the pathogenesis of the coronary artery stenosis and its severity in ACS patients. An antagonism was detected between the G allele at rs599839 and the exposure to high serum level of LDL-C, with calculated synergy index (*S*) of 0.41 (0.15–1.14) between the interaction terms as shown in Table 7. Furthermore, the interaction between the rare alleles of rs646776 and rs4970834 suggested the presence of antagonistic effect with an* S* 0.57 (0.21–1.54) and* S* 0.43 (0.15–1.27), but with a larger 95% CI, respectively.

## 5. Discussion

In this study, we examined the association among LDL-C, the coronary artery stenosis, and the 3 genetic variations rs599839, rs4970834, and rs646776 on the chromosomal locus 1p13.3. The study was carried on in a well-defined Arab population presenting with ACS. The main findings of this study are as follows: (1) the G allele carrier of the variant rs599839 had a significant protective effect against the risk of the significant atherosclerotic lesion in coronary arteries, (2) the significant association of polymorphism rs599839 (GG carriers) with lower LDL-C levels and total cholesterol levels, and (3) the antagonism between the G allele at rs599839 and the exposure to high serum level of LDL-C. Moreover, the C-allele carriers of rs646776 variant had a significant association with increased HDL-C. In addition, the interaction between high LDL-C with the genetic variants nearby SORT1 gene located in chromosomal locus 1p13.3 suggested the presence of antagonistic effect of the minor alleles, G, C, and T of rs599839, rs64776, and rs4970834 (Table 7), and by the GTC haplotype formed by the minor alleles of the studied SNPs (Table 6), respectively, on the significant stenosis of the coronary arteries as outcome of this interaction. The implications of these findings will be discussed in the following paragraphs.

The SNP rs599839 in* CELSR2-PSRC1-SORT1* on 1p13 revealed robust support for associations with LDL levels and risk of ACS in our study. The current finding for rs599839 is consistent with the results from previous studies in the Caucasian populations [14, 17, 33]. The GWAS of cardiovascular-related quantitative traits in the Framingham Heart Study by Roslin et al. (2009) reported associations between rs599839, on chromosome 1p13 and LDL [34]. A recent study by Zhou et al. (2011) demonstrated that rs599839 was significantly associated with CHD liability in a Chinese Han population with an effect on LDL through the risk A allele [35]. Sandhu et al. (2008) identified that rs599839 was associated with LDL levels using GAWS data from 11,685 European subjects [14]. Willer et al. (2008) described a correlation between rs599839 with both LDL levels and CHD risk in Caucasians [16]. Samani et al. (2008) reported an association of rs599839 with LDL cholesterol with myocardial infarction (*P* = 0.0026) [33]. The underlying mechanism through which rs599839, rs646776, and rs4970834 affect LDL-C is not well understood. One possibility is that these SNPs or associated genetic markers may influence the expression of* SORT1* gene that mediates the endocytosis and degradation of lipoprotein lipase [36]. Musunuru et al. (2010) have suggested a functional proof for this novel regulatory pathway for lipoprotein metabolism and proposed that any change might modify the hazard for myocardial infarction [37]. In addition, Angelakopoulou et al. (2012) stated that SORT1 rs599839 was correlated with the risk of CHD with odds ratio and 95% CI (1.20; 1.15–1.26) as well as total and LDL cholesterol and Apolipoprotein B [38].

In addition, our study demonstrated that G allele carriers of rs599839 had lower LDL-C and C allele carrier of rs646776 had an effect on increasing the HDL-C, which acts as scavenger lipoprotein that prevents the accumulation of lipid contacting particles with could predispose to plaque formation with high triglyceride levels. The current data is consistent with earlier results by Muendlein et al. (2009) which revealed that the rare alleles of variants rs599839, rs646776, and rs4970834 were significantly correlated with decreased serum LDL cholesterol (132 ± 40 mg/dL versus 125 ± 36 mg/dL, *P* = 0.003, 132 ± 40 mg/dL versus 124 ± 36 mg/dL, *P* < 0.001, and 131 ± 40 mg/dL versus 125 ± 37 mg/dL, *P* = 0.005), respectively [17].

However, the discrepancy between the findings of our study for rs4970834 and the previous results by Muendlein et al, 2009 [17], could be due to difference in ethnic background, population stratification, and sample size.

In addition, the current data provide further evidence of involvement of genetic variants rs599839 that could affect the LDL level among study subjects by multivariate regression analysis. The logistic regression analysis in our study showed that the variant rs599839 only could exert its effect independently from the other variants rs646776 and rs4970834, and such effect could be attributed to the low LD between these SNPs among our study subjects. Overall, the present data support the role of 1p13.3 locus on the level of LDL, which could play a role as a genetic variant in the pathogenesis of atherosclerotic in CAD patients.

The data presented here indicate a role of the chromosomal locus with the significant stenosis in all coronary arteries, in line with earlier studies [18, 33]. The finding of the current study supported the previous studies that showed an association between 1p13.3 locus and CAD [16, 33, 34, 37, 38]. Logistic regression analysis and haplotype estimation in the current study showed that the variant rs599839 exerted its effect on CAD independently and with associations of the other variants rs646776 and rs4970834. This may point to a major role of variant rs599839 in atherogenesis. This finding needs further investigation to clarify the mechanisms behind the association of rs599839 with atherogenesis in CAD patients.

The current study showed low pairwise LD values between the studies SNPs, which is different from other populations such as the Caucasian between rs599839 and rs646776 (*r* ^2^ = 0.51) (http://www.hapmap.org/) meaning that this locus has a different genetic structure in Arab population. In addition the current study showed that the G allele frequency in rs599839 (0.43), C allele of rs646776 (0.20), and T allele of rs49708934 (0.37) are different than that reported for other populations (http://www.hapmap.org/). This variation may be due to due to the difference in ethnic background, population stratification, and sample size. Such findings may explain the discrepancies in the genetic effect on diseases among different ethnic populations and need further studies.

Moreover, the current data showed an interesting finding that the G allele carriers of rs599839 variant decrease the severity of coronary stenosis using the dominant genetic models independent of the dyslipidemia. This does not primarily indicate that the A allele carriers of rs599839 polymorphism commence cardiovascular risk by itself individually from its impact on total cholesterol and LDL cholesterol levels. Definitely, a genotype, which is correlated with cholesterol and LDL cholesterol, may reveal previous and permanent exposure to LDL cholesterol better than the current LDL cholesterol levels themselves, particularly in CAD patients with the use of lipid-lowering and antidiabetic medication. This postulation is reinforced by the fact that LDL cholesterol serum concentrations were not significantly different between 2 groups in our study and statin and diabetes have no effect on the severity of stenosis in our ACS patients. This finding may suggest a strong relationship between the polymorphisms on chromosomal locus 1p13.3 and LDL cholesterol. The genetic polymorphisms at locus 1p13 and lipid profile may contribute in the same underlying pathogenesis that leads to atherosclerosis and CAD. This finding is consistent with previous reports indicating the decrease of LDL-C and total cholesterol in carriers of the minor allele G of rs599839 may decrease the severity of CAD and risk of MI among other ethnic populations [14, 32, 33, 36]. Furthermore, we evaluated this assumption by examination of the biological interaction between genetic markers at locus 1p13.3 and high serum LDL-C because they contribute to the same underlying pathogenesis that leads to atherogenesis and CAD. The findings of the current study found that studied variants at the locus 1p13.3 antagonize the risk of significant stenosis due to the exposure to high LDL-C. These data are in line with previous studies in GWAS [16, 33] and in SHEEP studies [32].

This study has several limitations; one of the most important is the sample size due to relatively small number of populations in Qatar. The selection of the studied SNPs to be explored in the current study relies on previously published data and does not include other tagged SNPs associated with the lipid profile such as rs611917 and rs12740374. The phenotypes under study (CAD stenosis and LDL-C level) are composite and might include different size effects of the polygenic/environmental/genes interactions. As such, the data may be biased and in addition to the small sample size of the current study may explain why other variants rs646776 and rs4970834 did not show significant associations with the significant stenosis of CAD in our study.

## 6. Summary and Conclusion

Polymorphism of rs599839 is significantly associated with LDL-C levels. In addition, the variant rs599839 has a significant association with the significant atherosclerotic lesion in coronary vessels. Moreover, polymorphisms of rs599839, rs646776, and rs4970834 at the locus 1p13.3 antagonize the risk of significant stenosis caused by the exposure to high LDL-C.

Future longitudinal studies in healthy populations are necessary among the Qatari people to explore the role of such locus and others variants for atherogenesis.

## Figures and Tables

**(a):** 

Variables	Group (A) *N* = 91	Group (B) *N* = 136	*P* value
Age (years)	56.86 (9.34)	56.33 (10.59)	0.701
BMI (kg/m^2^)	31.69 (6.19)	30.57 (6.94)	0.897
Body weight (kg)	81.85 (26.17)	80.49 (27.79)	
SBP (mmHg)	129.73 (16.38)	132.95 (21.84)	0.290
DBP (mmHg)	72.42 (10.21)	72.83 (9.95)	0.790
Glucose (mM)	6.94 (5.47–8.41)	9.49 (8.15–10.83)	0.003
LDL (mM)	2.69 (2.42–2.96)	2.81 (2.59–3.01)	0.510
HDL (mM)	1.01 (0.94–1.11)	0.97 (0.92–1.04)	0.551
TC (mM)	4.61 (4.25–4.97)	4.70 (4.19–5.22)	0.728
TG (mM)	2.58 (1.45–3.07)	1.96 (1.71–2.21)	0.568
Troponin (ng/mL)	2.42 (0.01–4.29)	3.53 (1.01–7.87)	0.130
CK-MB (ng/mL)	7.68 (0.75–44.43)	34.53 (4.63–141.35)	0.082

Continuous data are presented as means (SD) for normally distributed data and mean and lower and upper 95%, CI. Categorical variables were presented as number and percent. BMI: body mass index, SBP: systolic blood pressure, DSB: diastolic blood pressure, TC: total cholesterol, TG: triglycerides, LDL-C: low-density lipoprotein cholesterol, HDL-C: high-density lipoprotein cholesterol, CK-MB: creatinine kinase MB protein. Two-tailed *P* value is significant at <0.05.

**(b):** 

Variables	Group A Nonsignificant stenosis	Group B Significant stenosis	*P* value

CAD risk factors (*N*, %)	*N* = 91 (40%)	*N* = 136 (60%)	
Gender			
Male	68.0 (74.7%)	110.0 (80.9%)	0.269
Female	23.0 (25.3%)	26.0 (19.1%)	
Obesity	45.0 (49.5%)	71.0 (52.2%)	0.736
Hypertension (yes)	54.0 (59.3%)	84.0 (61.7%)	0.842
DM (yes)	40.0 (43.8%)	65.0 (62.5%)	0.008
Dyslipidemia (yes)	28.0 (33.73%)	41.0 (32.80%)	0.885
Smoking (yes)	30.0 (32.9%)	63.0 (46.3%)	0.062
FH of CAD (yes)	22.0 (24.1%)	40.0 (29.4%)	0.465
Use antidiabetic treatment	26.0 (28.6%)	40.0 (38.1%)	0.151
Use statin treatment	25.0 (34.2%)	38.0 (36.2%)	0.224

Categorical variables were presented as number and percent. DM: type 2 diabetes, FH of CAD: family history of coronary artery diseases. Two-tailed *P* value is significant at <0.05.

**Table 2:** Frequency of genotypes and alleles among study subjects based upon the significance of coronary artery stenosis stratification in CAD patients.

SNPs	Genotype	*N* (%)	Group A Insignificant stenosis	Group B Significant stenosis	*χ* ^2^	*P*
rs599839 A>G	AA	107 (48.4)	48 (56.5)	59 (43.4)	3.668	0.159
	AG	36 (16.3)	11 (12.9)	25 (18.4)		
	GG	78 (35.3)	26 (30.6)	52 (38.2)		

MAF	G (0.434)		G (0.371)	G (0.474)	3.682	0.032

rs646776 T>C	TT	170 (76.9)	73 (80.2)	97 (72.9)	1.568	0.456
	TC	18 (8.0)	6 (6.6)	12 (9.0)		
	CC	36 (16.1)	12 (13.2)	24 (18.1)		

MAF	C (0.201)		C (0.165)	C (0.236)	2.119	0.146

rs4970834 C>T	CC	122 (54.2)	48 (53.9)	74 (54.4)	4.147	0.125
	CT	40 (17.8)	11 (12.4)	29 (21.3)		
	TT	63 (28)	30 (33.7)	33 (24.3)		

MAF	T (0.369)		T (0.425)	T (0.316)	0.934	0.317

Data are presented by frequency for minor allele [MAF] and *P* value is significant at >0.05. Frequency of alleles is presented as a percentage. Data were analyzed by chi-square test (2 degrees of freedom) with Bonferroni adjustments. Two-tailed *P* value is significant at <0.05.

**Table 3 tab3:** Estimated marginal means (95% CI) of the lipid profile based upon the genotypes of the studied SNPs in all study subjects.

	TC	TG	HDL-C	LDL-C
rs599839 A>G-genotypes				
AA	5.29 (3.92–6.81)	3.02 (0.85–6.09)	1.44 (0.84–1.93)	3.44 (2.92–3.90)
AG+GG	5.06 (4.21–5.92)	2.87 (0.75–6.72)	1.48 (0.99–1.86)	2.92 (2.27–3.65)
GG	4.13 (3.62–5.05)	2.68 (0.46–7.58)	1.58 (0.89–2.72)	2.58 (2.31–3.39)
AA+AG	4.53 (3.77–5.38)	3.49 (0.78–7.66)	1.46 (0.78–1.91)	2.74 (2.00–3.47)
AG	4.66 (3.17–5.38)	3.08 (0.79–7.63)	1.52 (0.78–2.26)	2.68 (2.21–3.77)
*P* value for rs599839 genotypes				
GG vs. AA+AG	0.180	0.905	0.731	0.638
AG+GG vs. AA	0.210	0.604	0.630	0.050
GG vs. AA	0.041	0.628	0.751	0.026
rs646776 A>G-genotypes				
TT	4.62 (4.07–5.35)	6.69 (0.97–12.47)	1.15 (0.44–1.85)	2.93 (2.37–3.47)
TC+CC	4.46 (3.61–5.31)	2.42 (1.12–6.24)	2.04 (0.48–2.36)	2.79 (1.87–3.69)
CC	4.69 (3.38–6.02)	2.30 (1.34–12.31)	2.16 (1.54–2.78)	2.52 (1.73–3.30)
TC	4.52 (3.02–6.04)	3.54 (0.72–8.09)	1.44 (1.21–2.77)	2.53 (1.74–3.33)
TT+TC	4.61 (3.37–5.98)	4.52 (0.88–9.29)	1.36 (0.95–1.76)	2.64 (1.98–3.30)
*P* value for rs646776 genotypes				
TC+CC vs. TT	0.590	0.250	0.041	0.272
CC vs. TT+TC	0.972	0.087	0.021	0.252
CC vs. TT	0.906	0.018	0.004	0.427
rs4970834 C>T-genotypes				
CC	4.92 (4.12–5.72)	3.11 (1.18–8.16)	1.51 (0.94–2.07)	3.32 (2.48–4.15)
CT+TT	4.39 (3.66–5.13)	2.78 (1.69–7.69)	1.44 (1.02–1.87)	2.99 (2.36–3.63)
TT	4.19 (3.85–5.54)	2.42 (0.97–6.56)	1.38 (0.79–1.97)	2.74 (2.19–3.29)
CT	4.62 (3.45–5.81)	2.64 (0.77–6.98)	1.48 (0.92–1.98)	3.10 (2.45–3.75)
CC+CT	4.75 (3.62–5.87)	2.87 (1.02–7.15)	1.46 (0.86–2.01)	3.01 (2.47–3.73)
*P* value for rs4970834 genotypes				
CC vs. CT+TT	0.311	0.845	0.757	0.792
TT vs. CT+CC	0.880	0.852	0.657	0.165
CC vs. TT	0.752	0.912	0.812	0.386

Analysis of variance by general linear model with adjusted age, gender, BMI, type 2 diabetes, smoking, hypertension, and lipid-lowering therapy. LDL-C: low-density lipoprotein cholesterol and HDL-C: high-density lipoprotein cholesterol. Two-tailed *P* value is significant at <0.05.

**Table 4 tab4:** Stepwise regression analysis for LDL-C (dependent) and selected independent variables; age, gender, BMI, diabetes, and antilipidemic treatment (statin) and rs599839, rs646776, and rs4970834.

Independent variable	*β*-coefficient	Standard coefficient	Standard error	*P* value
Age	−0.016	−0.510	0.032	0.706
Gender	0.840	0.516	1.627	0.611
BMI	0.132	2.38	0.056	0.028
Diabetic state	−0.558	−0.240	0.168	0.005
Smoking	0.038	0.062	0.617	0.951
Statin	0.570	1.146	0.497	0.266
rs4970834	0.337	0.493	0.684	0.627
rs646776	0.065	0.020	0.246	0.791
rs599839	1.74	2.69	0.646	0.014

*P* < 0.05 is significant for levels of *β*-coefficient at 2-tailed testing.

**Table 5:** Results from logistic regression analysis: associations between determined SNPs and significant coronary stenosis for carriers of the rare allele compared to carriers of the homozygous common allele.

SNP	Model	OR	95% CI	*P* value
rs599839	Model 1	0.59	0.34–0.97	0.049
	Model 2	0.56	0.32–0.94	0.043
	Model 3	0.51	0.30–0.92	0.038

rs646776	Model 1	0.62	0.27–1.43	0.263
	Model 2	0.55	0.21–1.46	0.227
	Model 3	0.53	0.19–1.45	0.213

rs4970834	Model 1	0.98	0.57–1.68	0.944
	Model 2	0.83	0.45–1.51	0.545
	Model 3	0.88	0.46–1.70	0.705

Three logistic regression models were built: model 1 remained unadjusted, model 2 adjusted for age, gender, obesity, hypertension, diabetes, smoking, and family history of CAD, and model 3 for the covariates adjusted in model 2 and additionally LDL, HDL, and statin. OR, odds ratio, and CI, confidence interval. Two-tailed *P* value is significant at <0.05.

**Table 6 tab6:** Estimated haplotype frequency of the rs599839, rs646776, and rs4970834 polymorphisms in chromosomal locus 1p13.3 and its associations with the severity of coronary artery lesion in coronary artery disease (CAD) patients for significant stenosis versus nonsignificant stenosis outcome.

rs599839		rs4970834		rs646776		Haplotype	EM frequency ≥50%	EM frequency <50%	Adjusted OR, 95% Cl	*P* value
A	G	C	T	T	C					
A	G	C	T	T	C	ATC	0.284	0.364	0.69 (0.46–1.05)	0.090
A	G	C	T	T	C	GTC	0.295	0.178	0.54 (0.21–0.96)	0.005
A	G	C	T	T	C	ACC	0.186	0.219	0.82 (0.51–1.32)	0.410
A	G	C	T	T	C	GCC	0.093	0.135	0.66 (0.36–1.21)	0.172
A	G	C	T	T	C	GCT	0.061	0.034	1.85 (0.70–4.89)	0.208
A	G	C	T	T	C	ATT	0.043	0.058	0.73 (0.30–1.74)	0.471
A	G	C	T	T	C	GTT	0.028	0.007	4.00 (0.58–27.57)	0.128

Data are presented as frequency for severity of coronary artery lesion in CAD patients with significant lesion (≥50%) versus insignificant stenosis (<50%) for each haplotype with the odds ratio, lower and upper 95% confidence intervals, and *P* value. The data were analyzed by logistic regression analysis. ORs were adjusted for age, gender, BMI, type 2 diabetes, smoking, hypertension, and lipid-lowering therapy. Two-tailed *P* value is significant <0.05.

**Table 7:** Effect of interaction between serum LDL-C and genetic variants at Ip13.3 locus on the risk of significant coronary stenosis in ACS patients.

SNP	Model	OR	95% CI	Synergy index (*S*) with 95% CI
	High LDL	1.27	0.68–1.97	
rs599839	AG+GG vs. AA	0.59	0.34–1.02	
	Both	0.87	0.36–2.13	0.41 (0.15–1.14)

	High LDL	1.27	0.68–1.97	
rs646776	TC+CC vs. TT	0.62	0.27–1.43	
	Both	1.01	0.26–3.95	0.57 (0.21–1.54)

	High LDL	1.27	0.68–1.97	
rs4970834	CT+TT vs. CC	0.98	0.57–1.68	
	Both	1.04	0.45–2.40	0.43 (0.15–1.27)

Data are presented by OR and 95 % CI. Both means high LDL and genetic model effect on risk of stenosis, calculated by logistic regression analysis (see stat analysis). Synergy index (*S*) with 95% CI is biological measure of the interaction between two effects on one biological parameter, and if *S* below 1 means it is antagonistic and if above 1 suggests synergistic or additive.
